# FlavorMiner: a machine learning platform for extracting molecular flavor profiles from structural data

**DOI:** 10.1186/s13321-024-00935-9

**Published:** 2024-12-10

**Authors:** Fabio Herrera-Rocha, Miguel Fernández-Niño, Jorge Duitama, Mónica P. Cala, María José Chica, Ludger A. Wessjohann, Mehdi D. Davari, Andrés Fernando González Barrios

**Affiliations:** 1 Grupo de Diseño de Productos y Procesos (GDPP), Department of Chemical and Food Engineering, Universidad de los Andes, 111711 Bogotá, Colombia; 2 Leibniz-Institute of Plant Biochemistry, Department of Bioorganic Chemistry, Weinberg 3, 06120 Halle, Germany; 3 Institute of Agrochemistry and Food Technology (IATA-CSIC), Valencia, Spain; 4 Systems and Computing Engineering Department, Universidad de Los Andes, 111711 Bogotá, Colombia; 5 MetCore -Metabolomics Core Facility. Vice-Presidency for Research, Universidad de Los Andes, Bogotá, Colombia; 6 CasaLuker S.A, Bogotá, Colombia

**Keywords:** Flavor chemistry, Molecular machine learning, Molecular representation, Deep learning, Cocoa

## Abstract

Flavor is the main factor driving consumers acceptance of food products. However, tracking the biochemistry of flavor is a formidable challenge due to the complexity of food composition. Current methodologies for linking individual molecules to flavor in foods and beverages are expensive and time-consuming. Predictive models based on machine learning (ML) are emerging as an alternative to speed up this process. Nonetheless, the optimal approach to predict flavor features of molecules remains elusive. In this work we present FlavorMiner, an ML-based multilabel flavor predictor. FlavorMiner seamlessly integrates different combinations of algorithms and mathematical representations, augmented with class balance strategies to address the inherent class of the input dataset. Notably, Random Forest and K-Nearest Neighbors combined with Extended Connectivity Fingerprint and RDKit molecular descriptors consistently outperform other combinations in most cases. Resampling strategies surpass weight balance methods in mitigating bias associated with class imbalance. FlavorMiner exhibits remarkable accuracy, with an average ROC AUC score of 0.88. This algorithm was used to analyze cocoa metabolomics data, unveiling its profound potential to help extract valuable insights from intricate food metabolomics data. FlavorMiner can be used for flavor mining in any food product, drawing from a diverse training dataset that spans over 934 distinct food products.

**Scientific Contribution** FlavorMiner is an advanced machine learning (ML)-based tool designed to predict molecular flavor features with high accuracy and efficiency, addressing the complexity of food metabolomics. By leveraging robust algorithmic combinations paired with mathematical representations FlavorMiner achieves high predictive performance. Applied to cocoa metabolomics, FlavorMiner demonstrated its capacity to extract meaningful insights, showcasing its versatility for flavor analysis across diverse food products. This study underscores the transformative potential of ML in accelerating flavor biochemistry research, offering a scalable solution for the food and beverage industry.

**Supplementary Information:**

The online version contains supplementary material available at 10.1186/s13321-024-00935-9.

## Introduction

Flavor is defined as the combination between taste and odor, without distinction, of a substance or product [1]. This feature plays an essential role in defining consumer's acceptance of foods and beverages. The flavor properties of most processed food products are readily manipulable by formulating the proper ingredients to meet the desired sensory quality. Nonetheless, in the case of fermented products, matured foods, fruits, and vegetables, the flavor is purely determined by their chemical composition (including that of the matrix) [2]. The flavor molecules are produced in complex biological processes or chemical reactions and may be modified during processing, causing a pleasant or unpleasant sensation depending on their interaction with flavor receptors [2, 3]. Hence, understanding the impact of these compounds on the flavor profile is essential for engineering and decision-making in food and beverage production. This knowledge is critical for discovering and developing new flavors, standardizing and optimizing processing conditions, and variety selection to produce plant-based foods with a better flavor, among other applications [2, 4, 5].

The FlavorDB database (https://cosylab.iiitd.edu.in/flavordb/) is the most comprehensive collection of molecules with an experimentally validated flavor profile [6, 7]. This database also contains information from other flavor databases and external sources such as PubChem. Although this database contains more than 25,500 compounds, only 2254 metabolites (~ 9%) are linked to the 936 food products [6, 7]. In contrast, FooDB (https://foodb.ca/), the largest food metabolomics database, contains more than 24,000 compounds identified in foods [8]. The number of food-related molecules without flavor profiles will probably continue increasing in the future, boosted by the growing power of high-throughput metabolomics techniques and the complexity of testing the flavor profile of individual molecules. In addition, for most synthetic compounds no flavor profile is known.

The current experimental methodologies to assess the flavor profile of individual molecules require the compound to be isolated or synthesized [9]. Alternatively, the flavor is inferred by correlating metabolomics data with sensory results or tested using trained panelists [3, 10–12]. This procedure is considerably time-consuming and expensive, especially considering the complex composition of most food products. A recent alternative to streamline this process is the procedure called ultra-fast GC E-nose, which is an automated methodology to discriminate samples based on their odor fingerprint [13, 14]. The main drawback of this approach is that it does not assign a flavor profile to single compounds. Similarly, gas chromatography–olfactometry (GC-O) combines the power of gas chromatography with the sensitivity of the human nose to assign a flavor profile to individual compounds [12]. However, only volatile compounds can be tested following this technique, also perception even of an identical compound can be different when ingested orally in a matrix.

Similar to several other knowledge domains, different Machine Learning (ML) models have been developed to perform *in-silico* flavor prediction from molecular structures with available flavor profiles [3]. This trend has been prompted by advancements in ML algorithms and the availability of large-scale molecular data [15–17]. This approach enables an efficient screening of potential flavors to prioritize compounds for validation using traditional experimental methods, saving time and resources [2, 3, 18]. Nevertheless, ML models require diverse datasets to learn effectively. This variability is even more relevant for flavor prediction because molecules with similar structures can have completely different flavors or divergent molecular structures can have a similar flavor profile [3, 18]. Obtaining high-quality flavor data with sufficient coverage of different molecular structures can be challenging. In some cases, data may be limited or biased, leading to potential inaccuracies in predictions.

Most studies in this area used binary classifiers concentrated on predicting sweet and bitter flavors, mainly because of the availability of large datasets with labeled sweet and bitter molecules [16, 19–22]. These developments were also fostered by the need to address consumer preferences and health concerns. With increasing interest in reduced sugar consumption, the food industry is demanding alternative sweeteners or taste enhancers mimicking the desired tastes while reducing sugar content. Similarly, predicting bitterness or off-flavors is crucial for avoiding unpleasant taste experiences. Another reason boosting bitterness prediction is the wide prevalence of this flavor note in natural bioactive compounds, serving then as a potential screening tool [22–24]. Additionally, some predictors for sourness and umami have been published, although with less success because the availability of labeled data is limited for these flavor notes [19, 25].

The existing public flavor predictors cannot predict notes like floral, nutty, fruity, or off-flavor [22, 25, 26]. Some attempts have been undertaken in this area, but the performance is still poor and the code and data are not publicly available [3, 27–29], leading to a lack of available multilabel tools for flavor prediction. These flavor notes are of capital importance in fermented food processing (including coffee, beer, wine, chocolate, bread and others) [2]. Also, some models based on Generative Artificial Intelligence have been trained to generate new molecules with flavors potentially interesting for the food industry (including the above-mentioned flavors), but they lack any classification capability [4, 17]. A major challenge for predicting these flavor notes is the class imbalance, as the number of positive examples is significantly lower than the negatives [2, 25]. Even though some class balancing methods are available (e.g., over-sampling and under-sampling) [30, 31], it is unclear what approach works better for flavor prediction.

The most widespread approaches for flavor prediction are based on Quantitative Structure–Activity Relationship (QSAR) models, which have been extensively used in drug discovery [2, 3, 18, 32]. These models correlate the chemical structure and properties of molecules with their biological activity, including flavor. The main ML algorithms used have been Random Forest, Support Vector Machines, K-Nearest Neighbors, Deep Neural Networks [20], and more recently Graph Neural Networks [2, 18, 19]. These models use mathematical features that capture molecular properties, such as atomic composition, connectivity, and physicochemical properties [18]. Molecular descriptors followed by molecular fingerprints are the most widespread mathematical representation of molecular structure to train flavor predictors [2, 18, 20]. Recently, molecular graph representations were also implemented for flavor prediction [4, 18, 19]. However, to date, no study comparing the performance of these mathematical representations has been reported.

In response to the identified gaps, the present study aimed to develop FlavorMiner, a flexible and retrainable flavor ML predictor for seven flavor categories critical for quality control within the food industry (i.e., floral, fruity, sour, sweet, bitter, off-flavor, and nutty). This research comprises an exploration of the performance of different mathematical representations of molecular structures and classification algorithms for flavor prediction. Demonstrating its prowess, FlavorMiner was applied successfully to cocoa metabolomics data, showcasing its ability to unlock valuable insights from food metabolomics data. It can be utilized for flavor analysis in various food products, thanks to a diverse training dataset encompassing over 934 different food products.

## Results and discussion

**Fig. 1:**
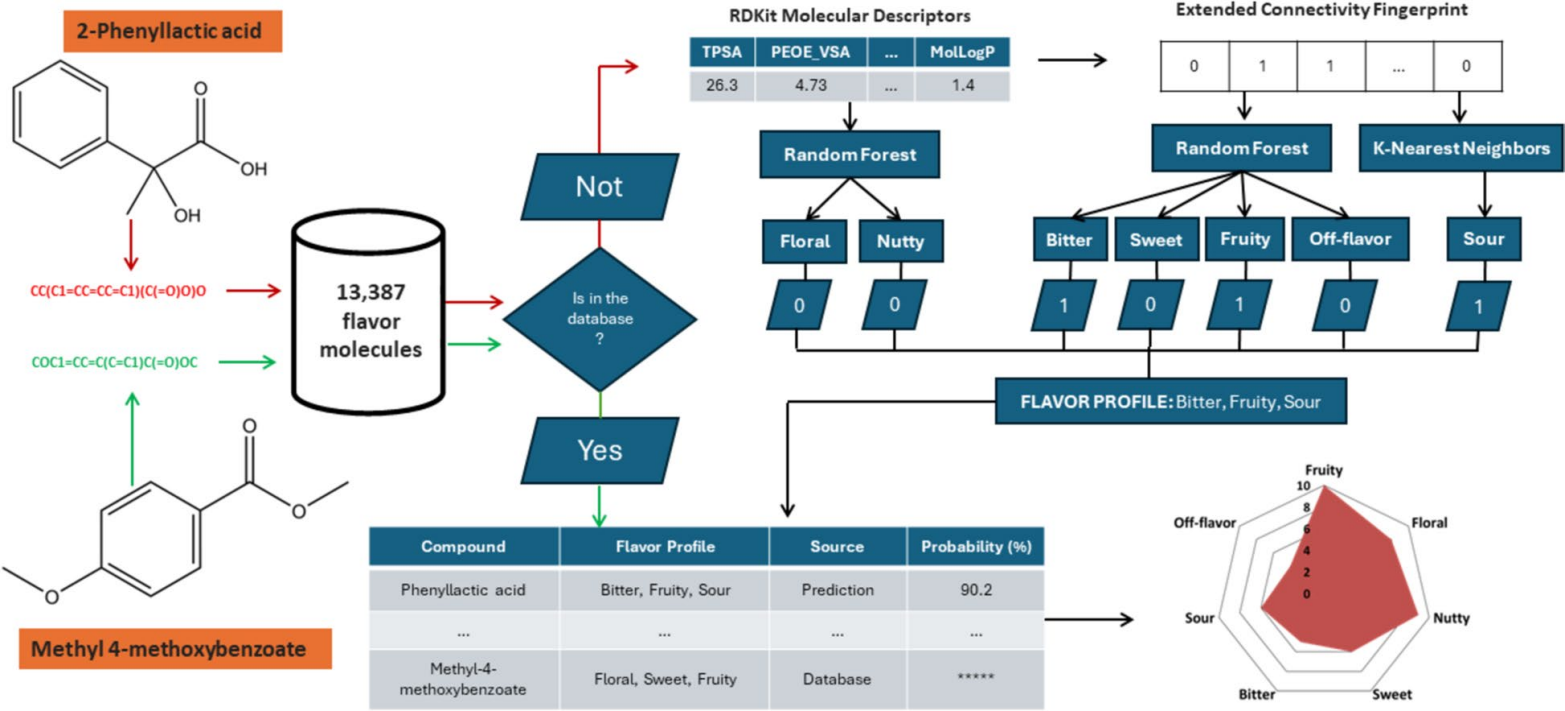
Workflow of the FlavorMiner algorithm for flavor prediction and data visualization. The algorithm requires input compounds structured in Isomeric SMILES format, essential for both database matching and prediction steps

In this work we introduce the FlavorMiner algorithm, which takes as input the Isomeric Smiles of a set of molecules and produces as output their flavor profile (Fig. 1). The first step is to query a database of 13,387 molecules with known flavor profiles. Only the set of molecules with no database match pass to the prediction step. Then, the respective mathematical representation of the molecules is generated. In the next step, this mathematical representation is fed to seven independent binary classifiers. The average prediction capability of these predictors is 0.88 (ROC AUC Score). Each classifier predicts one of the seven target flavor categories (bitter, floral, fruity, off-flavor, nutty, sour, and sweet). The results are provided in a table, including the predicted flavor profile for each compound and the source of the flavor profile (database match or prediction). The probability values are also provided, indicating the confidence level of each prediction. Finally, a radar chart showing the recurrence of the molecules with each target flavor is also generated.

### Development of ML models for flavor prediction, including management of class imbalance biases

**Fig. 2:**
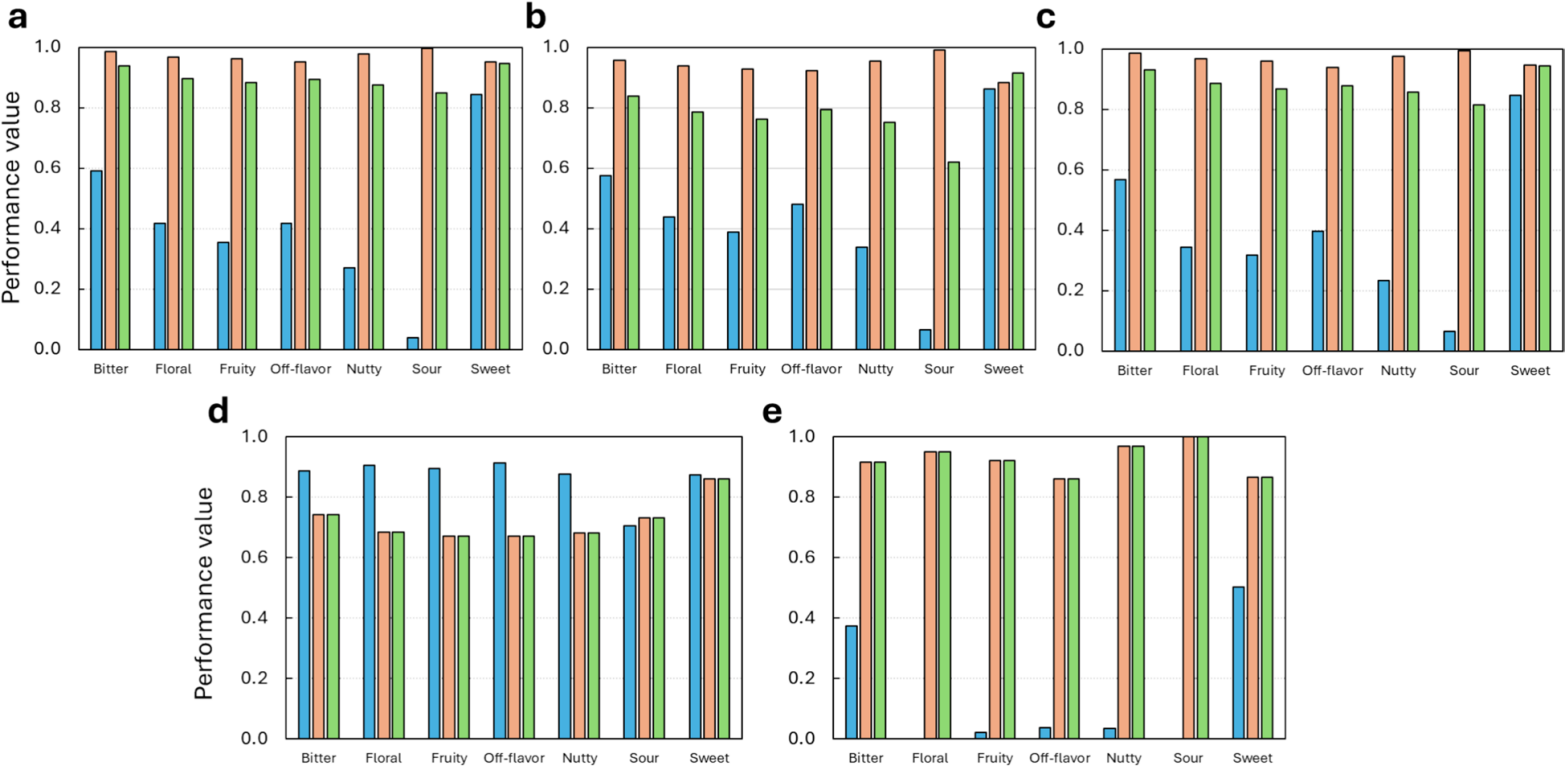
Classification Metrics for Algorithms Trained with Original Descriptor Data on Test Set. The metrics include Recall (blue bar), Specificity (orange bar), and ROC AUC Score (green bar) for each algorithm. **a** Random Forest trained with molecular descriptors. **b** Random Forest trained with extended connectivity fingerprint. **c** K-Nearest Neighbors trained with molecular descriptors. **d** K-Nearest Neighbors trained with extended connectivity fingerprint. **e** Convolutional Graph Neural Network trained with molecular graph

To train the classifiers incorporated in FlavorMiner, a flavor molecule dataset was assembled, containing 13,387 compounds with experimentally validated flavor profiles. The positive examples (those with a specific flavor) represent on average 20% of the dataset, while the negative examples (those without a specific flavor) represent 80% (Supplementary Fig. 1). This is a sign of class imbalance, which is an important challenge in the development of ML models, as it can lead to bias towards the majority class [33, 34]. Due to this class imbalance, all algorithms trained on the original data, except for those trained on sweet molecules, had poor recall (Fig. 2), which measures the ability to correctly identify positive examples [30, 31]. On the other hand, the specificity, which measures the ability to correctly identify negative examples [30, 31], was significantly high. This bias towards the majority class was consistently observed regardless of the target flavor, algorithm, or mathematical representation.

Most algorithms trained on the original data showed a specificity higher than 0.9 during the test. Nonetheless, these models had a recall lower than 0.5. This performance evidence a bias towards the majority class higher than 40% for most algorithms. The Convolutional Graph Neural Network trained with the original molecular graph had the lowest recall for most target flavors (close to zero) (Fig. 2c). This is likely because this algorithm is more complex (larger number of parameters) and hence it requires more data to be trained effectively [35, 36]. On the other hand, the sweet category has a bias of less than 10% with Random Forest and K-Nearest Neighbors, trained either with RDKit descriptors or ECFP, which can be explained because it had the smallest class imbalance. The number of sweet positives is only 2% lower than the negative examples. Conversely, the sour category has a class imbalance of 97% and showed the highest bias towards the majority class (> 85%). This is a common issue in ML models dealing with imbalanced data [37].

Additionally, overfitting was observed in models using Random Forest and K-Nearest Neighbors after the first training iteration with the original data. This is likely due to the limited number of positive data, which can lead the model to exceedingly consider specific features from the negative training set that constrain the ability to adequately generalize and predict when presented with previously unseen data [30, 38, 39]. The difference between the train and test specificity was under 10% in most cases, but the recall showed a considerable drop between 20 and 90% from train to test for most algorithms trained with the original data (Supplementary Fig. 2). This is a clear sign of overfitting [30]. Only some models obtained with the Convolutional Graph Neural Network showed no sign of overfitting. This is only because the recall was near to zero both during training and testing. Similarly, there was a proportional relationship between class imbalance and overfitting. For example, the models for the sweet flavor (the class with no imbalance) showed the lowest overfitting, while the sour flavor (the class with a high imbalance and special problems associated with its perception, v.i.) had the highest overfitting percentage.

SMOTE and Cluster Centroid sampling techniques were implemented to address the class imbalance. These strategies significantly reduced bias and overfitting. SMOTE, an oversampling technique previously used in flavor predictors [19, 20, 28], was applied to the minority class to increase the number of positive examples. This resulted in a bias of less than 20% for most algorithms (Supplementary Fig. 3). The overfitting level was also reduced to less than 30% for most algorithms (Supplementary Fig. 4). Under-sampling with Cluster Centroid [40] was also applied to reduce the number of negative examples (Supplementary Fig. 5). This resulted in an overfitting reduction to less than 30% for K-Nearest Neighbors models and less than 15% for Random Forest models (Supplementary Fig. 6). Most K-Nearest Neighbors models had a bias of less than 10%, while most Random Forest models had a bias of over 20%.

Bias and overfitting were reduced due to a significant increase in the recall after applying the resampling strategies. Although the bias and overfitting were still slightly high, this represented a significant improvement compared to the performance with the original data. The recall of all the algorithms trained with resampled data was over 50%, while the specificity of most of these models remained above 70%. Multiple studies have shown that both oversampling and undersampling can be used to correct the problems caused by class imbalance in machine learning approaches [33, 34, 40]. In the context of flavor prediction, several studies have investigated the effect of SMOTE oversampling [19, 20, 28]. These studies have focused mainly on sweet, bitter, and sour flavors, and have obtained results similar to those of the present work [19, 25, 38].

On the other hand, using a balancing transformer on the molecular graph to train a convolutional graph neural network significantly improved the recall, but also significantly reduced the specificity. The recall for classes with more class imbalance improved by 73–99%, but the specificity dropped by a similar proportion (Supplementary Fig. 7). Additionally, the recall for classes such as sweet and bitter decreased. Consequently, the bias and overfitting increased for all models trained with the balanced molecular graph compared to the original data. The bias was higher than 50% for most target flavors and was as high as 90% for fruity, off-flavor, nutty, and sour flavors. This indicates that the balancing transformer had a significant negative effect on the specificity of the models. The overfitting for bitterness and sweetness predictions increased with the balanced data. For fruity, off-flavor, nutty, and sour, the recall decreased to negative values by more than 20%. A negative recall change value indicates underfitting, which occurs when the model does not learn a strong enough pattern from the training data [30, 31]. This can be solved by performing a more intense hyperparameter optimization, but this may come at a considerable computational cost compared to Random Forest and K-Nearest Neighbors algorithms.

The balancing transformer and resampling techniques (SMOTE and cluster centroid) differ in how they address class imbalance. The balancing transformer focuses on the weights of positive and negative examples in the neural network, while resampling techniques focus on the feature space [30, 40, 41]. The balancing transformer does not change the input data or the number of examples in each class [30]. The poor results obtained with this strategy demonstrate that this is insufficient to solve the severe class imbalance of the input data. Resampling techniques, on the other hand, change the input data by creating new synthetic examples in the minority class (SMOTE) or by removing examples from the majority class and replacing them with cluster centroids [40]. Considering the significant improvement in the performance of the algorithms trained with resampling techniques, this seems to be the best approach to balance the flavor compound database. Unfortunately, it is challenging to implement resampling strategies on molecular graphs, and only possible with molecular descriptors and fingerprints. This is because clustering molecular graphs without affecting their structure and losing valuable information is nearly impossible. Also, in flavor studies, minor changes in structure (graphs) can cause severe changes in perception. Thus, synthetic filling can cause more rather than fewer problems. Although other balance methods are available for graph data, their usefulness with molecular graphs remains to be evaluated [41].

### FlavorMiner combines the best ML models for prediction of different flavor classes.

Random Forest outperformed the K-Nearest Neighbors algorithm for most target flavor notes, except sour (see below for discussion). Random Forest trained with ECFP oversampled with SMOTE performed best for bitter, fruity, sweet, and off-flavor notes. Random Forest trained with RDKit descriptors performed best for floral and nutty notes. K-Nearest Neighbors trained with ECFP oversampled with SMOTE performed best for sour notes. In general, K-Nearest Neighbors had similar recall to Random Forest with the same input datasets, but slightly lower specificity. Also, algorithms trained with data resampled with the cluster centroid algorithm had slightly better recall, but a higher drop in specificity compared to datasets resampled with SMOTE. These results are consistent with previous studies, which found that Random Forest outperforms other algorithms for predicting sweet and bitter flavors [3, 22, 26]. A correlation was observed between the amount of positive data available and the performance of the classifiers. Sweet, the class with the highest number of positive instances, had the best overall performance, with an ROC AUC score of 0.97. Sour, the class with the lowest number of positive instances, had the lowest performance, with a ROC AUC score of 0.78. These results suggest a superior performance of algorithms trained with resampled datasets compared to those trained with the original data.

**Fig. 3:**
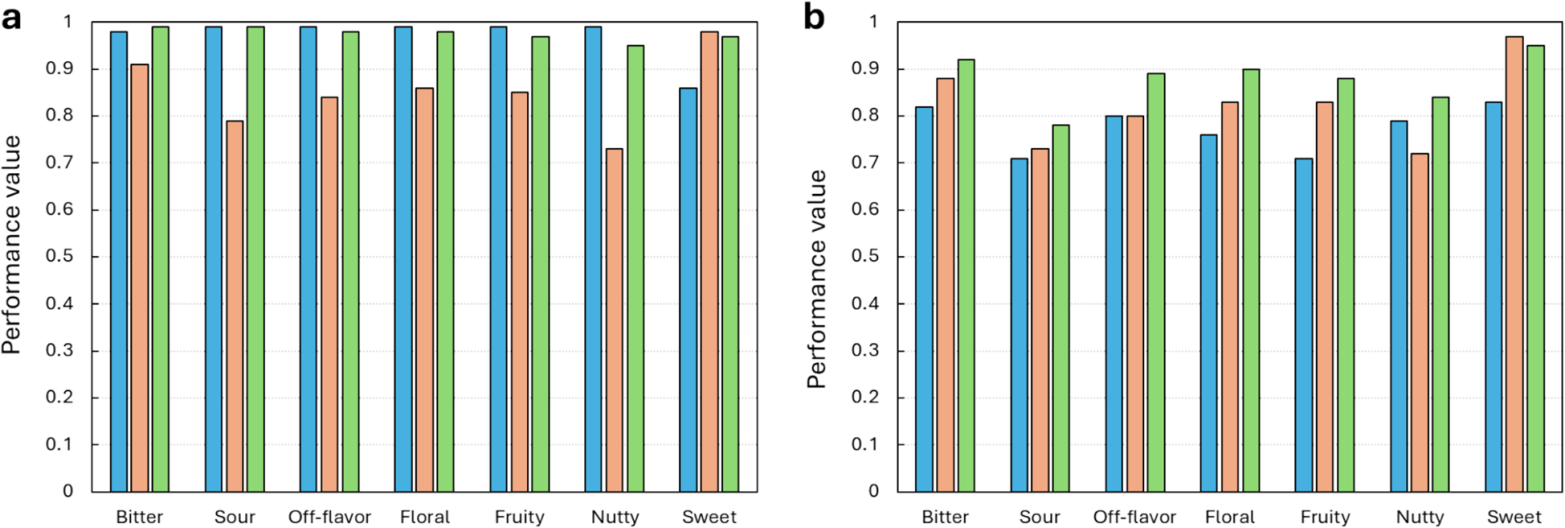
Performance of the Optimized Classifiers for Target Flavor Notes in FlavorMiner. The metrics include Recall (blue bar), Specificity (orange bar), and ROC AUC Score (green bar) for each algorithm. **a** Classification metrics obtained during training using fivefold cross-validation. **b** Classification metrics obtained using the test set. Random Forest was used for bitter, fruity, sweet, off-flavor, floral and nutty. K-Nearest Neighbors for sour notes

The performance of the seven final predictors selected for the FlavorMiner backbone is shown in Fig. 3. The average ROC score, specificity, and recall of these classifiers were 0.88, 0.82, and 0.77, respectively. The performance of FlavorMiner for bitter and sweet prediction was comparable to that of existing predictors [20, 22, 26]. For fruity and floral prediction, FlavorMiner achieved recalls of 0.71 and 0.76, respectively, representing an improvement of over 50% compared to previous studies [18, 42]. FlavorMiner is the first model to predict nutty and off-flavor notes from molecular structures. For sour prediction, FlavorMiner was outperformed by a previously published tool [25] by about 15%. However, the dataset, composition of positive and negative examples, and code used in this study are not publicly available, making it difficult to assess the reasons for the observed difference.

Variable importance plots (VIP) scores [31] revealed the most important features for predicting floral, off-flavor, and nutty notes (Supplementary Fig. 8) with RDKit molecular descriptors. Six descriptors appeared repeatedly in all three cases, accounting for around 45% of the classification. These descriptors measure properties such as the size and polarity of molecules (TPSA), their electronic structure (PEOE_VSA and EState_VSA) and stability (SMR_VSA1 and MinEStateIndex), and their tendency to partition into a hydrophobic environment (MolLogP). Supplementary Fig. 9 shows the trend of the five most relevant features for positive and negative examples of each flavor note. Off-flavor molecules tend to be smaller and less polar than non-off-flavor molecules, with a higher tendency to partition into hydrophobic environments. Floral molecules tend to be smaller and more flexible than non-floral molecules, with a higher tendency to partition into hydrophilic environments. Finally, nutty molecules tend to be smaller and less flexible than non-nutty molecules, with a higher electronic stability. These results are new for these flavor notes and provide a basis for future research to select more specific mathematical representations and use data mining techniques to better understand why molecules have these flavors.

**Fig. 4:**
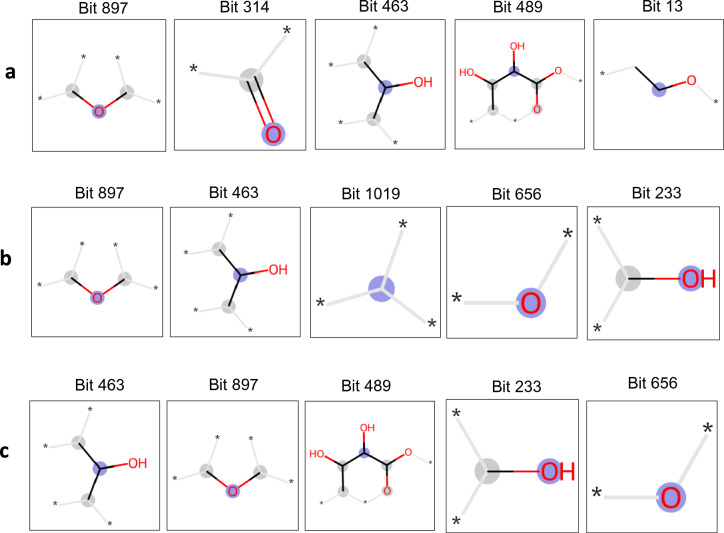
Fragments Representing High VIP Scores in Random Forest Predictors with ECFP. This figure displays fragments corresponding to the 5 bits with the highest Variable Importance (VIP) scores in Random Forest predictors trained with Extended Connectivity Fingerprint (ECFP). **a** Bitter, **b** Fruity, **c** Sweet

Supplementary Fig. 10 shows the VIP scores for the Random Forest models trained on oversampled ECFP descriptors for predicting bitterness, fruitiness, and sweetness. The four most important bits for the binary classifiers predicting these flavor notes were 897, 314, 489, and 463. The fragments corresponding to these bits are shown in Supplementary Fig. 4. For the K-Nearest Neighbors algorithm, the permutation importance score [43] was used to estimate feature importance because in this case it is not possible to use the VIP score (Fig. 4). Interestingly, most of the top five fingerprints for these notes corresponded to fragments that were absent in the positive compounds. This is likely due to the higher chemical diversity of the negative compounds. E.g. many typical bitter compounds contain an (alkaloid) nitrogen, but no N-containing fragment appeared in the top 5 for bitter, actually many top fragments like bit 897 (C–O–C—moiety) appeared in bitter, fruity and sweet, i.e. they are of universal flavor relevance, but for note determination probably play there role obviously only in the context with other features (e.g. in esters for fruity or sweet in cyclic sugars). Even though resampling strategies were implemented to improve the overall performance of the models, this did not necessarily enhance the chemical diversity of the positive examples.

Fingerprints have two main advantages over molecular descriptors. First, they can provide information about the structural features that lead an algorithm to a certain decision. Future work could involve a deeper analysis of the fragments that play a central role in the classification to better understand the structural features that underlie these flavor notes. Second, they can be calculated from MS-spectra data, even when the structure of a compound is not fully elucidated [44, 45]. This makes fingerprints useful for accelerating the discovery of new flavor molecules in metabolomics experiments. Metabolomics experiments typically involve the analysis of many compounds and ECFP can help concentrate the annotation and structural elucidation on the most promising candidates. This can save time and money by focusing efforts on the most promising and likely flavor-active compounds.

The CGNModel combined with molecular graphs showed poor performance, even with a balancing transformer. This is likely due to the inherent noisiness of the data, which is exacerbated by the susceptibility of Graph Neural Networks to noisy data [46, 47]. This noisiness arises from the heavy dependence of flavor characterization on human tasters and the influence of genetic, sensory, and environmental factors on flavor perception [3, 16, 48, 49]. It is challenging to implement a denoising strategy without losing valuable information. Therefore, the CGNModel was discarded for FlavorMiner, given the limitations of the current data and the better performance of other algorithms.

The flavor profile of a molecule also depends on its concentration and the surrounding matrix [50, 51]. This is related to the concept of flavor threshold and the synergistic and antagonistic effects of flavor molecules in complex mixtures. The flavor threshold is the minimum concentration at which the flavor is detectable [50, 51]. This version of FlavorMiner only performs binary prediction, and intensity data is not yet incorporated. Although some data is available, it is not readily accessible, as there is no standardized database of threshold concentrations for molecules with known flavor profiles. Some databases such as FlavorDB [6, 7] and LSB@TUM Odorant Database (https://www.leibniz-lsb.de/en/databases/leibniz-lsbtum-odorant-database/start/) contain information on flavor thresholds. However, there is a lack of standardization in the thresholds reported in these databases. This means that a method is needed to unify and make this data comparable. Also, most information on flavor thresholds is available in unstructured format (text). Therefore, an intense text mining process is required to extract this data and make it usable for machine learning purposes.

Additionally, some studies have shown that combining several molecules with different flavor profiles can enhance the flavor profile of a mixture or block certain notes [50, 51]. However, data in this area is limited, and any effort in this direction will require a preliminary experimental process to generate it. Overcoming these challenges could lead to the development of regression algorithms that can be combined with flavor classifiers to predict not only the flavor profile of a molecule but also its threshold concentration and matrix effect.

Sour (like salty not evaluated here) is a special flavor note, as it relies on the smallest available “molecule”, the proton. Also, it is not activating a classical GPCR like the other other taste (T1R and T2R) or the olfactory receptors. Only quite recently the responsible Otop1 ion channels were assigned [52]. Thus typical structural features of a molecule might be considered irrelevant, except for its pKa properties, i.e. its ability to lower pH, an effect that will strongly depend on the matrix’ overall pH, buffer capacity and may be the proton relay/ion transport capacity. Thus, predicting sour taste from structure might be considered impossible if only the pH change is sensed. However, like GPCRs, ion channels can be influenced by more than the ion it is selective for for various reasons, including ion pairing and matrix/mucosa effects or directly at the ion channel by secondary interactions and additional binding sites which will have selective structural preferences as every protein does. In conclusion, structure-based predictions for ion channel-based tastes (here sour, but also salty) have to be considered with utmost caution, as slight changes in the tasting parameters, e.g. of the matrix (pH, buffer capacity) can thwart results and thus all ML. To understand, if there is sour taste influence on the anionic, organic (i.e. structurally influenced) part, such taste experiments must run with a standardized, high-capacity buffered matrix, neutral pH or better at 2–3 different pH values. Only this can reveal any possible structural influences of the organic counterion or a neutral molecule influencing or mimicking sour taste. Otherwise, it will not be better than a standard pKa prediction which does not require ML. Independent of this, perception is also influenced by the other receptors. A classic example is of course the action of Miraculin.

### Molecular flavor prediction for compounds involved in the processing of cocoa

Previous studies have annotated around 210 compounds during the fermentation, drying, and roasting of fine-flavor cocoa [53, 54]. However, for less than half of these compounds a flavor profile has been reported. The exisiting data were analyzed with FlavorMiner to predict the flavor profile of these compounds. After the prediction, the compounds with “known” flavor profiles increased to 92%. The newly predicted compounds include 12 floral, 8 fruity, and 4 compounds with unknown fine-flavor attributes that are potentially linked to positive impacts on quality and price. Additionally, 2 compounds linked to off-flavors and 27 unknown potentially sweet compounds were suggested by the model. These predictions represent an important step forward in closing the gap between cocoa metabolic fingerprint variation during processing and flavor quality.

**Fig. 5:**
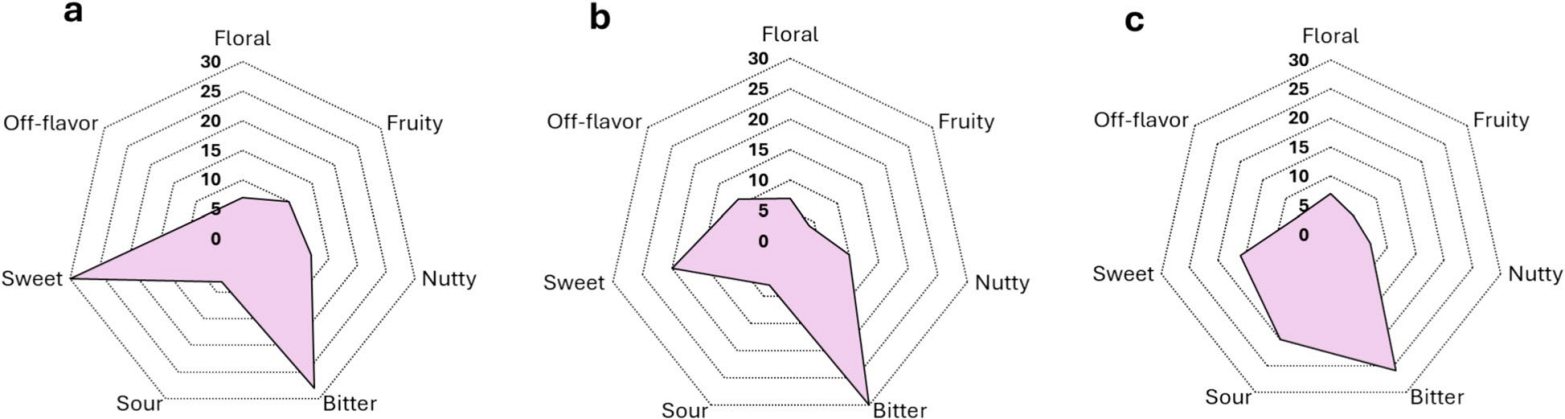
Molecule counts for each target flavor across cocoa processing stages (**a**) Fermentation, (**b**) Drying, (**c**) Roasting

Figure 5 shows the frequency of compounds increasing in association with each of the seven target flavors at the end of every cocoa processing stage (Fermentation, Drying, and Roasting). In general, the frequency of compounds for the different target flavors is similar during fermentation and drying. The most relevant change through the processing chain is in sweet compounds, which decrease considerably during the process. This drop is associated with a decrease in the carbohydrate content during the processing chain [53, 54], as most of these molecules are reported as sweet agents. In the roasted samples some compounds linked to sour and bitter showed a higher abundance, but the real impact of these suggested flavor molecules still needs to be elucidated. For example, some degradation products of more complex compounds have a lower biological activity (e.i., antioxidant activity) than their precursors [55]. If a similar trend occurs with respect to flavor will require further investigations. In contrast, most compounds linked to fine flavor notes (fruity, floral, and nutty) show a relatively constant frequency throughout the cocoa processing chain. These results provide further suggestions into flavor development from biochemistry to processing, which was a missing component until now.

## Conclusions

The present work led to the development of FlavorMiner, an open-access flavor ML predictor for fruity, floral, off-flavor, nutty, sour, sweet, and bitter notes. Access sites are: https://github.com/ipb-halle/FlavorMiner. This fills a gap in the current landscape of flavor prediction tools, as there are few open-access options available for predicting these specific flavor notes. FlavorMiner has an average ROC AUC score of 0.88. This algorithm performs similarly to other bitterness and sweetness predictors but outperforms existing floral and fruity predictors. It is also the first tool to predict nutty and off-flavor notes. This work also showed the superiority of RDKit descriptors and ECFP over molecular graphs (combined with Convolutional Graph Neural Network) as mathematical representations to predict flavor notes from molecular structures. Molecular graphs had a recall lower than 0.5 in most cases, even after balancing the classes with a transformer. This means that they currently fail to accurately identify molecular structures with the target flavors. On the other hand, models trained with RDKit descriptors and ECFP had a recall over 0.6 in most cases, especially after resampling with the SMOTE algorithm.

The best performers were selected to assemble the backbone of FlavorMiner, reaching a recall of over 0.7 in most cases. Further analyses of the best performers revealed the following properties that can define a molecule as positive or negative for floral, off-flavor, and nutty notes such as the size, polarity, electronic structure, stability, and their tendency to partition into a hydrophobic environment. Similarly, this analysis revealed the most critical fragments to define as positive or negative in a molecule for bitter, fruity, sour, and sweet, whereby predictions for sour have to considered with special caution. These results offer a solid base for future studies aiming to dissect the chemistry of flavor properties.

Using FlavorMiner with cocoa metabolomics data highlighted its potential to elucidate the molecular basis of flavor development in processed food products. To gain further insights into the chemistry and pharmacology of flavor, additional functionalities such as flavor threshold and interaction prediction should be included. However, several challenges must be overcome, starting with (unified) data availability. It is also important to note that machine learning techniques should complement traditional experimental methods in flavor prediction. Integrating the strengths of both approaches can enhance the overall accuracy and efficiency of flavor prediction and lead to new discoveries in the field.

## Materials and methods

### Data collection and preprocessing

The data for this study were collected from multiple sources, including FlavorDB, Sigma-Aldrich catalog, BitterSweet, ChemTastesDB, Flavornet, Off-flavor, AromaDB, OlfactionBase, and Natural_tAS2r_agonists [16]. The data were cleaned by removing duplicate entries, molecules without reported flavor profiles, invalid PubChem identifiers or names, molecules with fewer than 2 heavy atoms, and salts, knowing that especially the latter fact may depent on representation (in ionic or non-ionic form) of some molecules, which may also have contributed to the problems with the sour set. The linear structures of the remaining molecules were retrieved from the PubChem database using the Python library PubChemPy. The compounds were labeled with the flavor information retrieved from the databases. The flavor notes were grouped into seven categories: bitter, floral, fruity, off-flavor, nutty, sour, and sweet. The labeling process was automated in a Python script and a flavor wheel [56] to facilitate future relabeling or adding new data. The seven target labels were then converted into binary values using the One Hot Encoding method [30]. This information was stored in an Excel file containing the compound name, PubChem ID, flavor profile, isomeric smiles, data source, and labels (Supplementary File 1).

### Mathematical representation of molecular structures

Three mathematical representations of the molecular structures were generated. First, 200 molecular descriptors were calculated using the RDKit library. Descriptors with invalid data, more than 97% of unique values, and descriptors highly correlated were removed. For this, the Pearson correlation index was used with a threshold of 0.95 [57]. Second, an Extended Connectivity Fingerprint (ECFP) with a radius of 2 was generated for all compounds using the RDKit library. Fingerprints with more than 97% unique values were also removed. The datasets corresponding to the RDKit molecular descriptors and ECFP were split into training and testing sets using a random partition of 80:20. A third mathematical representation was generated by creating molecular graphs from the molecular structures using the DeepChem library. The DeepChem MolGraphConvFeaturizer was used to generate the molecular graphs. The graphs were then labeled for each target flavor with a binary label and converted into NumPy datasets. This dataset was divided into a training, validation, and testing set using a random partition of 70:10:20.

### Machine learning algorithms training, optimization, and testing

Independent binary classifiers were used to predict each flavor category. Initially, a Random Forest and K-Nearest Neighbors algorithm were trained on the RDKit molecular descriptors and ECFP datasets using scikit-learn. Hyperparameter optimization was performed using the Grid Search method and fivefold cross-validation. Class imbalance was addressed by oversampling the minority classes using SMOTE (Synthetic Minority Oversampling Technique) and undersampling the majority classes using the cluster centroid of a KMeans algorithm. A Graph Convolutional Neural Network (GCNModel) was then trained on the molecular graphs using DeepChem. Hyperparameter optimization of the GCNModel was performed using the hyperopt library. The balance of the molecular graph data was done using the DeepChem balancing transformer. The performance of the algorithms was evaluated using the recall, specificity and Receiving Operator Characteristic (ROC) curves [30, 31].

### Using cocoa processing metabolomics data as a case study

Two previous datasets from metabolomics studies of fine-flavor cocoa processing were used for this case study [53, 54]. The first study used LC-QTOF-MS and GC-QTOF-MS to analyze the fermentation of fine-flavored cocoa [53]. The second study used the same analytical platforms to assess the evolution of flavor during post-fermentation processing (drying and roasting) of fine-flavor cocoa [54]. The PubChem ID for each molecule was obtained from the PubChem database. This data were stored in an Excel file and fed to FlavorMiner to assign flavor profiles.

## Supplementary Information

Supplementary file 1.

## Data Availability

All the datasets, and scripts used in this work are publicly available in a GitHub repository (https://github.com/ipb-halle/FlavorMiner). FlavorMiner and all Python scripts are available in Jupyter Notebooks. These Jupyter Notebooks contain a detailed explanation of the code and the data, which is integrated with the GitHub repository, reducing the need to download files locally to run the scripts.
