# Supplementary material for: FlavorMiner: a machine learning platform for extracting molecular flavor profiles from structural data
Source: J Cheminform. 2024 Dec 10;16:140. doi: 10.1186/s13321-024-00935-9 (PMC11633011; doi:10.1186/s13321-024-00935-9)

SUPPLEMENTARY INFORMATION

FlavorMiner: unraveling the flavor profile of individual molecules from their structure with Machine Learning techniques.

Fabio Herrera-Rocha, Miguel Fernández-Niño, Jorge Duitama, Mónica P. Cala, María José Chica, Ludger A. Wessjohann, Mehdi D. Davari*, Andrés Fernando González Barrios*

Table of contents

Supplementary Figure 1. 2
Supplementary Figure 2. 3
Supplementary Figure 3. 4
Supplementary Figure 4. 5
Supplementary Figure 5. 6
Supplementary Figure 6. 7
Supplementary Figure 7. 8
Supplementary Figure 8. 8
Supplementary Figure 9. 10
Supplementary Figure 10. 11
Supplementary Figure 11. 12
Supplementary Figure 12. 12

Supplementary Figure 1. Distribution of Positive and Negative Examples in Molecular Flavor Datasets. This figure illustrates the distribution of negative and positive examples in the molecular flavor dataset across all target flavors. The legend also denotes the average number of compounds (Avg). The negative set consists of molecules lacking a specific flavor profile, while the positive set comprises molecules containing the flavor note. Additionally, (*) indicates the presence of an unclassified set containing molecules with flavor profiles not aligning with the target flavor notes.

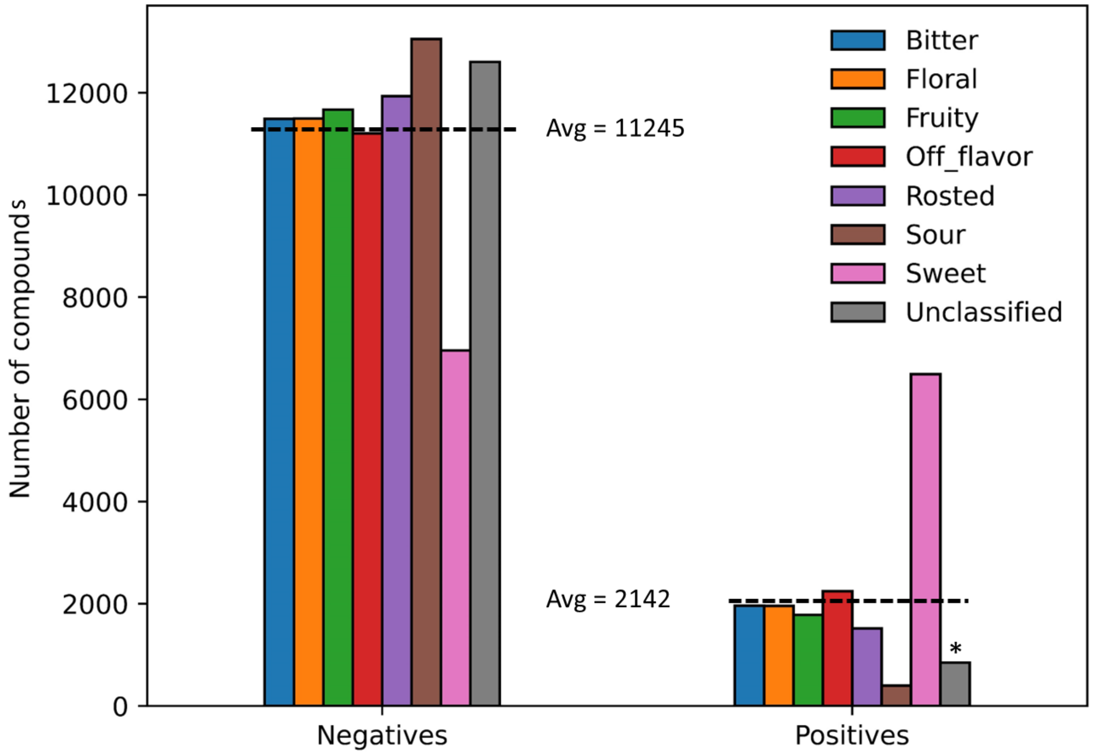

Supplementary Figure 2. Comparative Performance of Machine Learning Algorithms for Flavor Prediction during training. This figure illustrates the performance comparison of various machine learning algorithms for predicting seven target flavors during training using 5-fold cross-validation. Each bar represents Recall (blue bar), Specificity (orange bar), and ROC AUC Score (green bar) for the algorithms across the seven flavors. **(a)** Random Forest with RDKit Molecular Descriptors **(b)** K-Nearest Neighbors with RDKit Molecular Descriptors **(c)** Random Forest with RDKit Extended Connectivity Fingerprint **(d)** K-Nearest Neighbors with RDKit Extended Connectivity Fingerprint **(e)** Graph Neural Network with Molecular Graph.

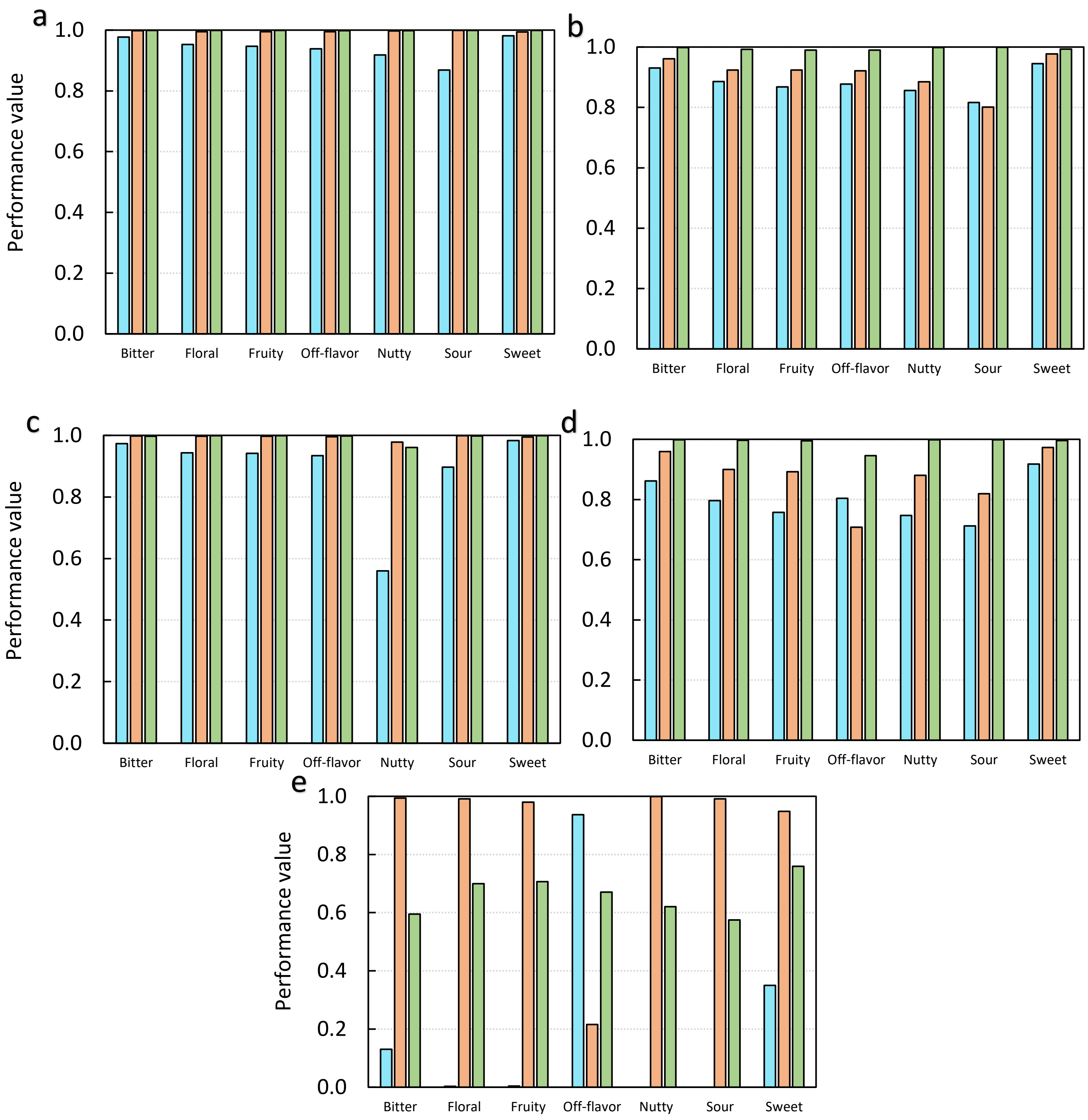

Supplementary Figure 3. Comparative Performance of Machine Learning Algorithms for Flavor Prediction using oversampled data. This figure illustrates the performance comparison of various machine learning algorithms for predicting seven target flavors during test using data oversampled with Synthetic Minority Over-sampling Technique (SMOTE). Each bar represents Recall (blue bar), Specificity (orange bar), and ROC AUC Score (green bar) for the algorithms across the seven flavors. **(a)** Random Forest with RDKit Molecular Descriptors **(b)** K-Nearest Neighbors with RDKit Molecular Descriptors **(c)** Random Forest with RDKit Extended Connectivity Fingerprint **(d)** K-Nearest Neighbors with RDKit Extended Connectivity Fingerprint.

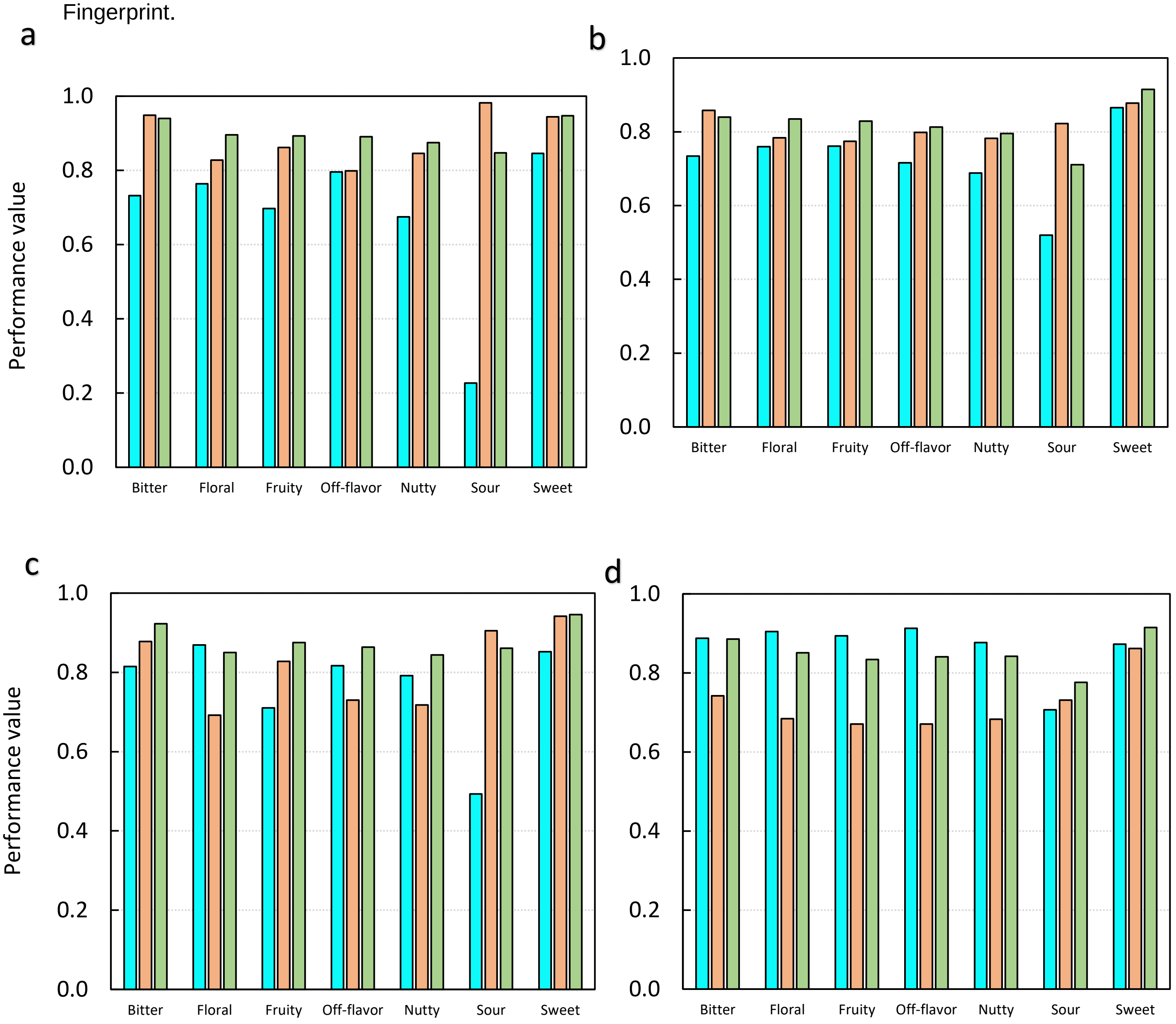

Supplementary Figure 4. Comparative Performance of Machine Learning Algorithms for Flavor Prediction using oversampled data during training. This figure illustrates the performance comparison of various machine learning algorithms for predicting seven target flavors during test using data oversampled with Synthetic Minority Over-sampling Technique (SMOTE) during training using 5-fold cross-validation. Each bar represents Recall (blue bar), Specificity (orange bar), and ROC AUC Score (green bar) for the algorithms across the seven flavors. **(a)** Random Forest with RDKit Molecular Descriptors **(b)** K-Nearest Neighbors with RDKit Molecular Descriptors **(c)** Random Forest with RDKit Extended Connectivity Fingerprint **(d)** K-Nearest Neighbors with RDKit Extended Connectivity Fingerprint.

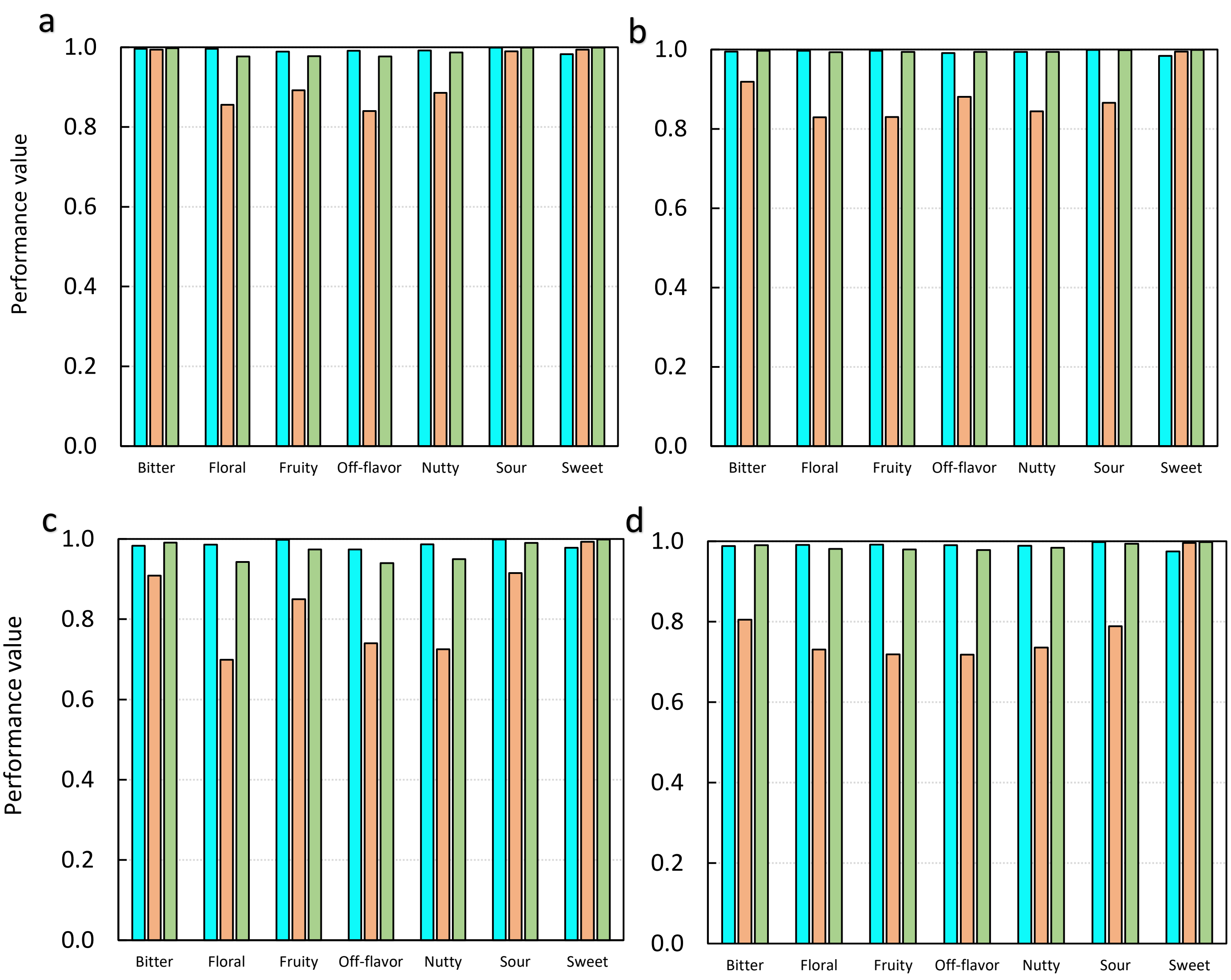

Supplementary Figure 5. Comparative Performance of Machine Learning Algorithms for Flavor Prediction using undersampled data. This figure illustrates the performance comparison of various machine learning algorithms for predicting seven target flavors during test using data oversampled with Cluster Centroid Technique. Each bar represents Recall (blue bar), Specificity (orange bar), and ROC AUC Score (green bar) for the algorithms across the seven flavors. **(a)** Random Forest with RDKit Molecular Descriptors **(b)** K-Nearest Neighbors with RDKit Molecular Descriptors **(c)** Random Forest with RDKit Extended Connectivity Fingerprint **(d)** K-Nearest Neighbors with RDKit Extended Connectivity Fingerprint.

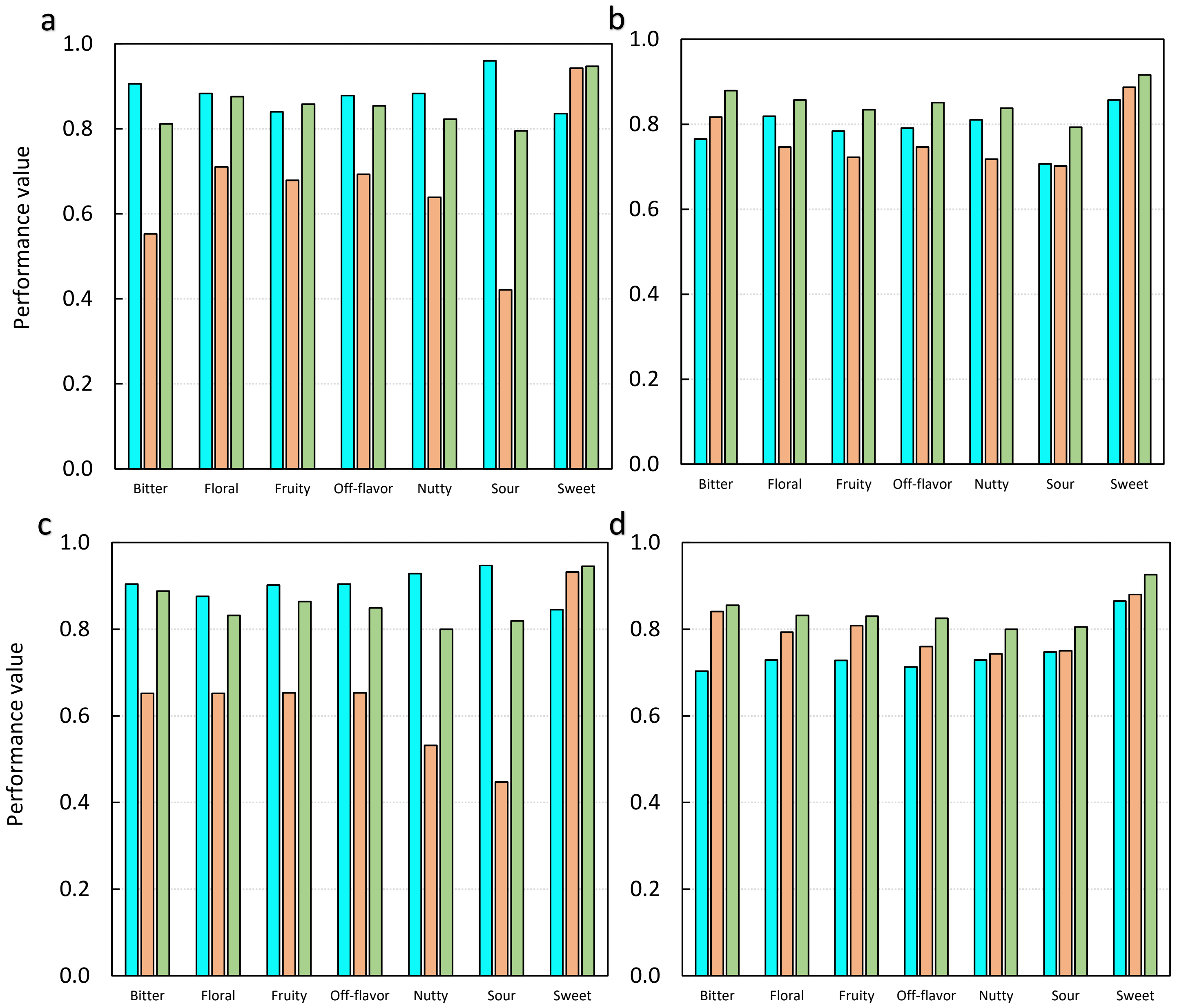

Supplementary Figure 6. Comparative Performance of Machine Learning Algorithms for Flavor Prediction using undersampled data during training. This figure illustrates the performance comparison of various machine learning algorithms for predicting seven target flavors during test using data undersampled with Cluster Centroid Technique during training using 5-fold cross-validation. Each bar Recall (blue bar), Specificity (orange bar), and ROC AUC Score (green bar) for the algorithms across the seven flavors. **(a)** Random Forest with RDKit Molecular Descriptors **(b)** K-Nearest Neighbors with RDKit Molecular Descriptors **(c)** Random Forest with RDKit Extended Connectivity Fingerprint **(d)** K-Nearest Neighbors with RDKit Extended Connectivity Fingerprint.

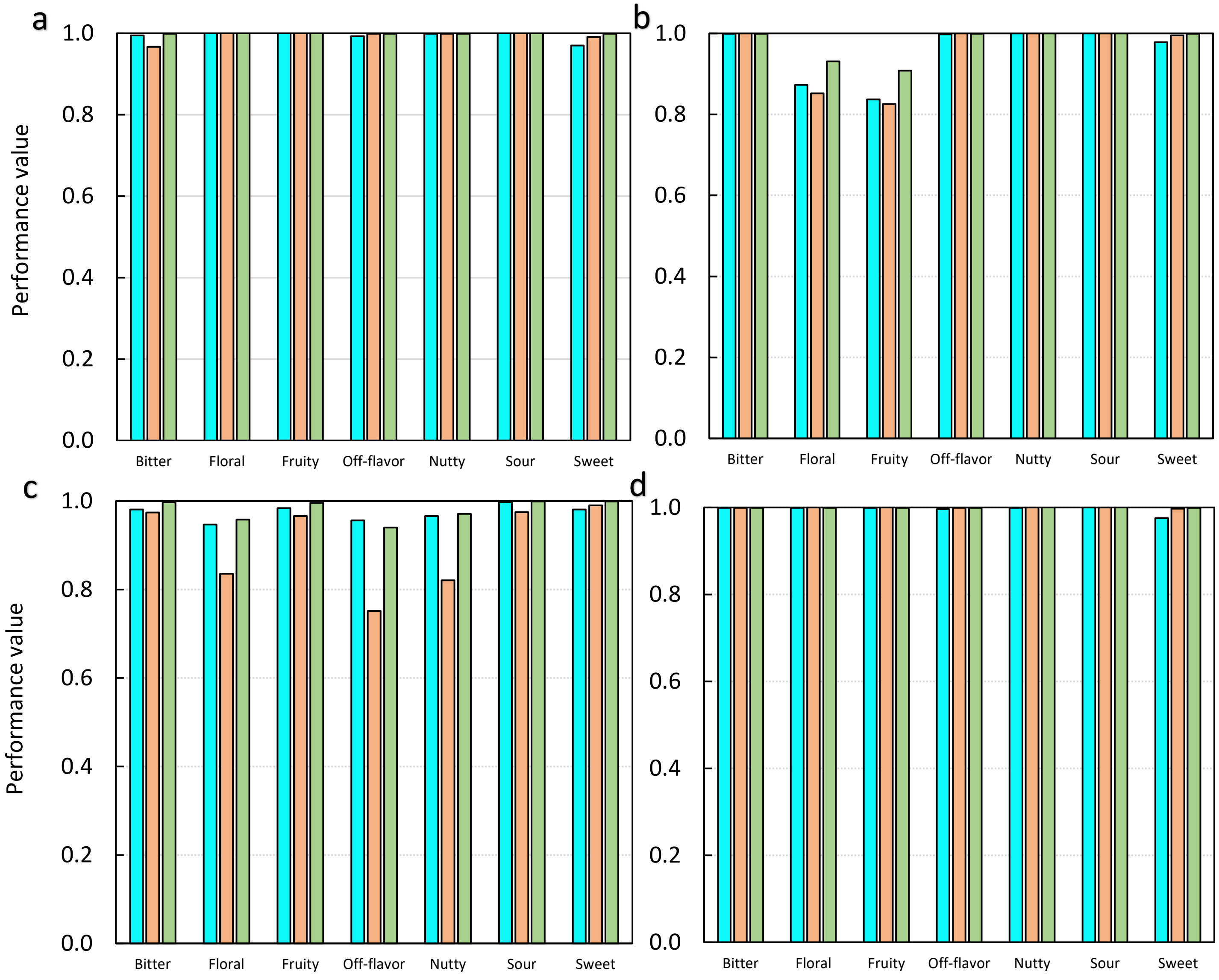

Supplementary Figure 7. Comparative Performance of Machine Learning Algorithms for Flavor Prediction using a Graph Neural Network. This figure illustrates the performance comparison of various machine learning algorithms for predicting seven target flavors during test using data oversampled with resampled Molecular Graph data. Each bar represents Recall (blue bar), Specificity (orange bar), and ROC AUC Score (green bar) for the algorithms across the seven flavors. **(a)** performance during test **(b)** performance during training.

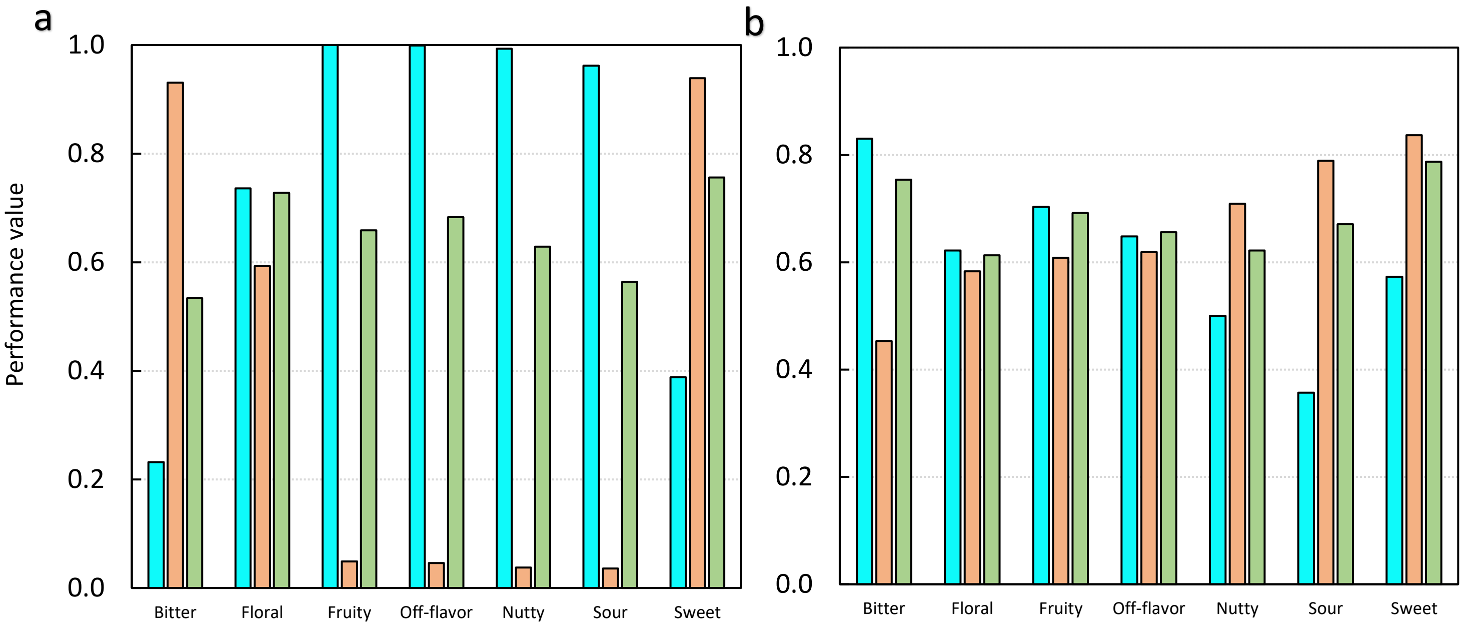

Supplementary Figure 8. Variable Importance Plot (VIP) for Random Forest Predictors with RDKit Molecular Descriptors. This figure showcases VIPs representing the final Random Forest predictors trained with RDKit molecular descriptors. **(a)** Off-flavor **(b)** Floral **(c)** Nutty. For an explanation of each descriptor go to RDKit documentation (http://www.rdkit.org/docs/GettingStartedInPython.html#list-of-available-descriptors).

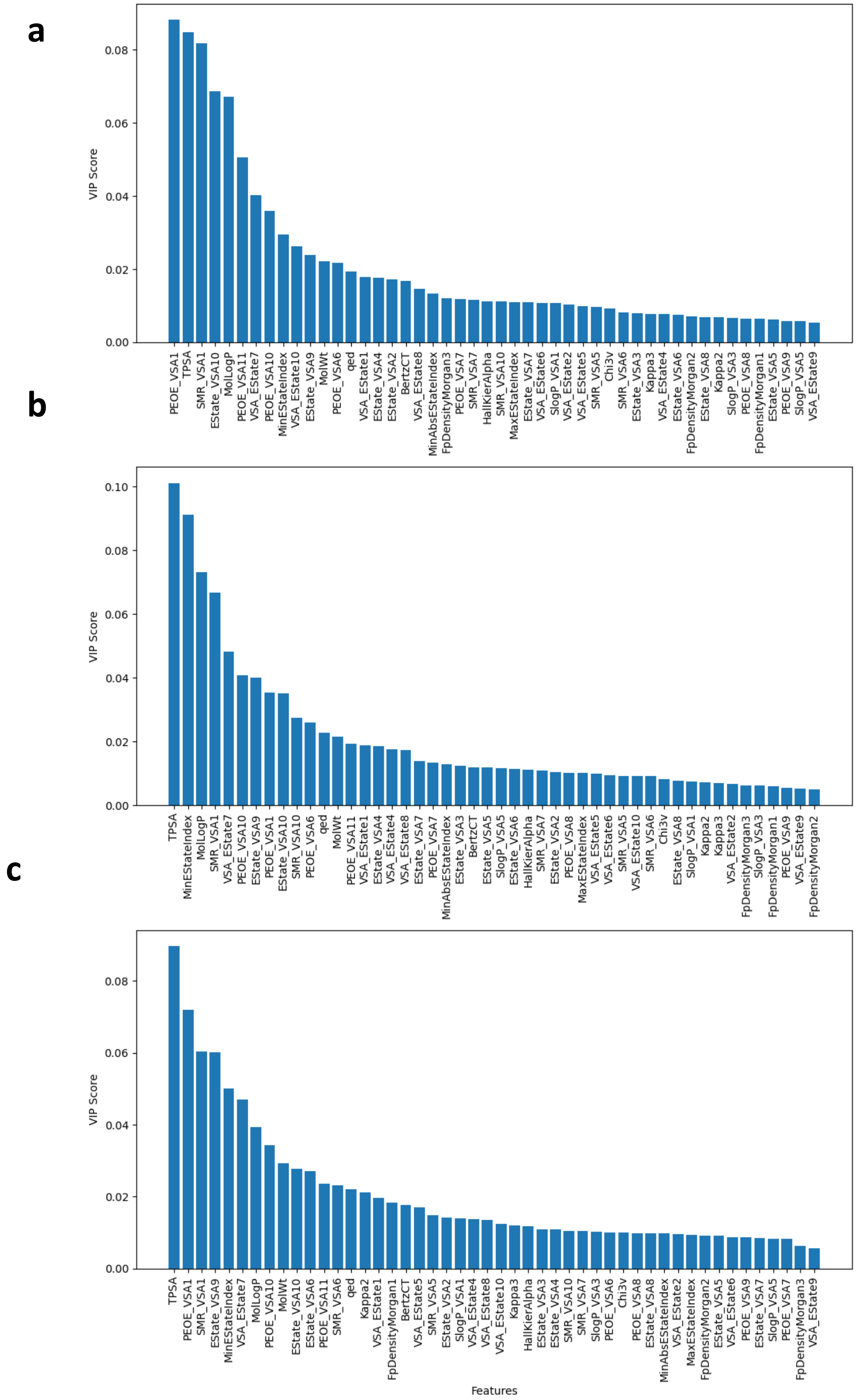
a
VIP Score
0.00
0.02
0.04
0.06
0.08
PEOE_VSA1
TPSA
SMR_VSA1
EState_VSA10
MolLogP
PEOE_VSA11
VSA_EState7
PEOE_VSA10
MinEStateIndex
VSA_EState10
EState_VSA9
MolWt
PEOE_VSA6
qed
VSA_EState1
EState_VSA4
EState_VSA2
BertzCT
VSA_EState8
MinAbsEStateIndex
FpDensityMorgan3
PEOE_VSA7
SMR_VSA7
HallKierAlpha
SMR_VSA10
MaxEStateIndex
EState_VSA7
VSA_EState6
SlogP_VSA1
VSA_EState2
VSA_EState5
SMR_VSA5
Chi3v
SMR_VSA6
EState_VSA3
Kappa3
VSA_EState4
EState_VSA6
FpDensityMorgan2
EState_VSA8
Kappa2
SlogP_VSA3
PEOE_VSA8
FpDensityMorgan1
EState_VSA5
PEOE_VSA9
SlogP_VSA5
VSA_EState9
b
VIP Score
0.00
0.02
0.04
0.06
0.08
0.10
TPSA
MinEStateIndex
MolLogP
SMR_VSA1
VSA_EState7
PEOE_VSA10
EState_VSA9
PEOE_VSA1
EState_VSA10
SMR_VSA10
PEOE_VSA6
qed
MolWt
PEOE_VSA11
VSA_EState1
EState_VSA4
VSA_EState4
VSA_EState8
EState_VSA7
PEOE_VSA7
MinAbsEStateIndex
EState_VSA3
BertzCT
EState_VSA5
SlogP_VSA5
EState_VSA6
HallKierAlpha
SMR_VSA7
EState_VSA2
PEOE_VSA8
MaxEStateIndex
VSA_EState5
VSA_EState6
SMR_VSA5
VSA_EState10
SMR_VSA6
Chi3v
EState_VSA8
SlogP_VSA1
Kappa2
Kappa3
VSA_EState2
FpDensityMorgan3
SlogP_VSA3
FpDensityMorgan1
PEOE_VSA9
VSA_EState9
FpDensityMorgan2
c
VIP Score
0.00
0.02
0.04
0.06
0.08
TPSA
PEOE_VSA1
SMR_VSA1
EState_VSA9
MinEStateIndex
VSA_EState7
MolLogP
PEOE_VSA10
MolWt
EState_VSA10
EState_VSA6
PEOE_VSA11
SMR_VSA6
qed
Kappa2
VSA_EState1
FpDensityMorgan1
BertzCT
VSA_EState5
SMR_VSA5
EState_VSA2
SlogP_VSA1
VSA_EState4
VSA_EState8
VSA_EState10
Kappa3
HallKierAlpha
EState_VSA3
EState_VSA4
SMR_VSA10
SMR_VSA7
SlogP_VSA3
PEOE_VSA6
Chi3v
PEOE_VSA8
EState_VSA8
MinAbsEStateIndex
VSA_EState2
MaxEStateIndex
FpDensityMorgan2
EState_VSA5
VSA_EState6
PEOE_VSA9
EState_VSA7
SlogP_VSA5
PEOE_VSA7
FpDensityMorgan3
VSA_EState9
Features

Supplementary Figure 9. Quartile Box Plot of Variable Importance for Models with RDKit Molecular Descriptors. This figure presents Quartile Box Plots illustrating the importance of the first 5 features in the Variable Importance Plot for models trained with RDKit molecular descriptors. Each subplot focuses on a specific flavor category. **(a)** Off-flavor, **(b)** Floral, **(c)** Nutty. Positive examples are denoted in green, while negative examples are represented in red. (For abbreviations like PEOE_VSA1 etc. see Suppl. Fig. 8)

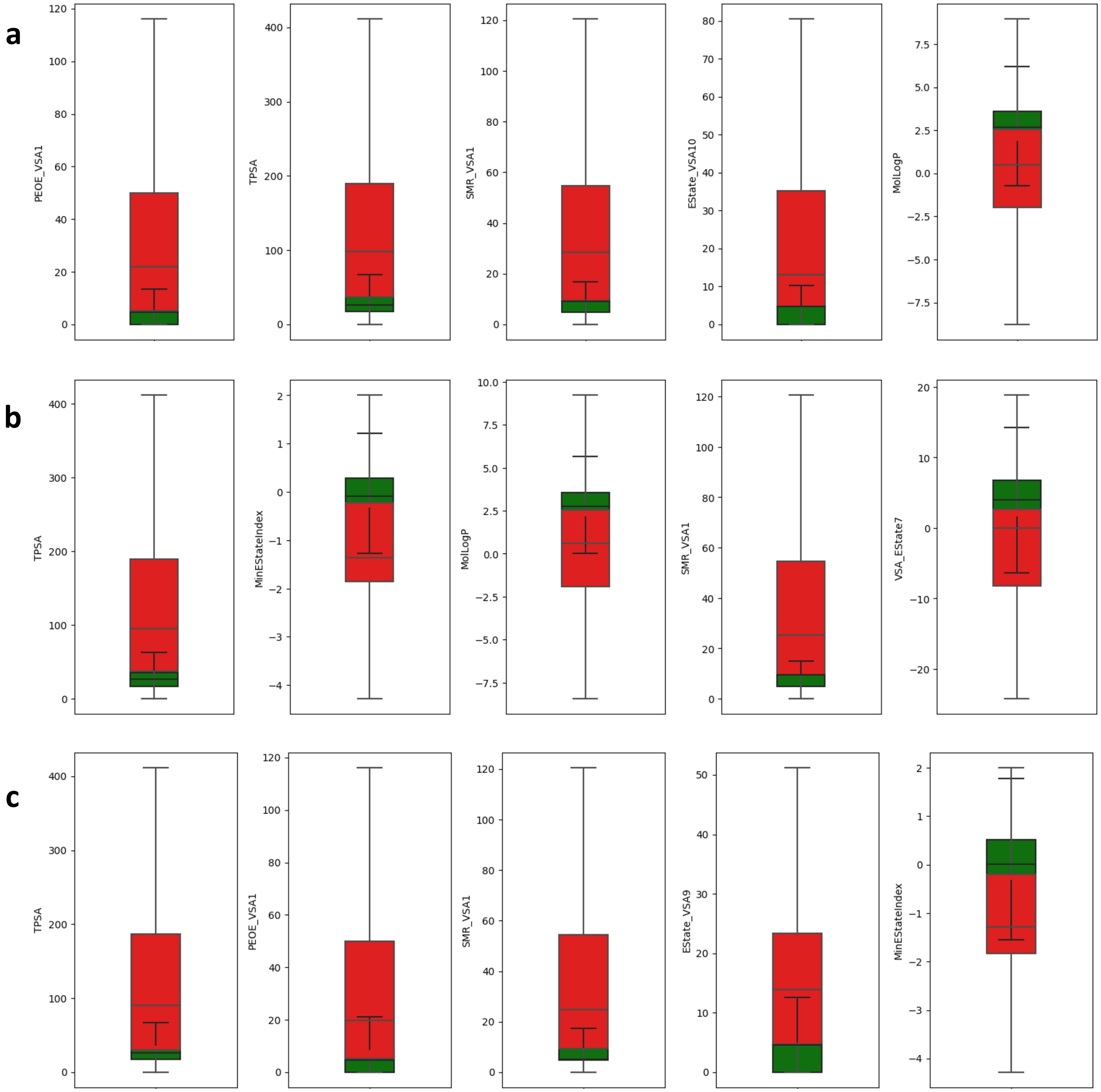

Supplementary Figure 10. Variable Importance Plot (VIP) for Random Forest Predictors with RDKit Molecular Descriptors. This figure showcases VIPs representing the final Random Forest predictors trained with Extended Connectivity Fingerprints (ECFP). **(a)** Off-flavor **(b)** Floral **(c)** Nutty

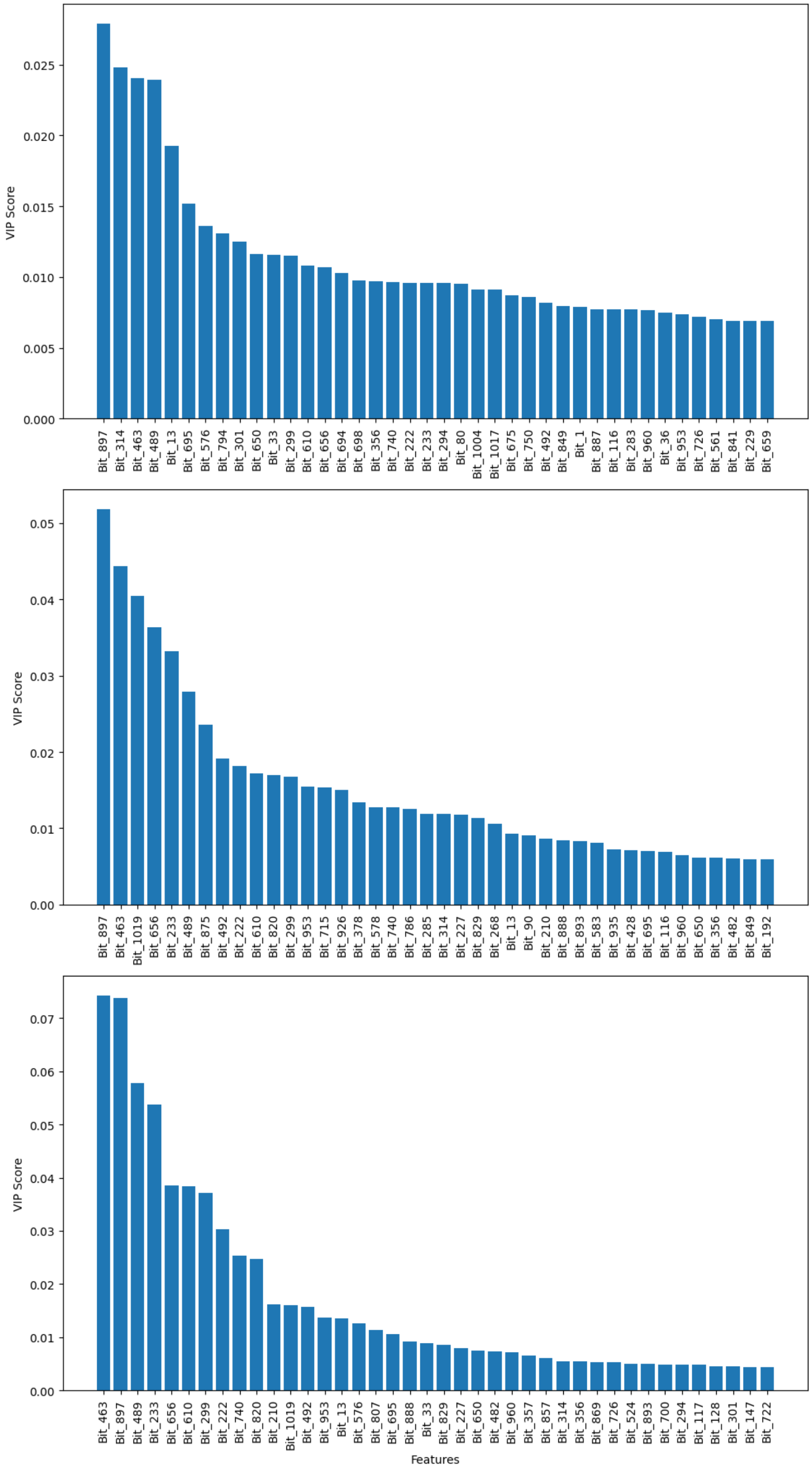

Supplementary Figure 11. Permutation Score Plot specifically for the Sour predictor.

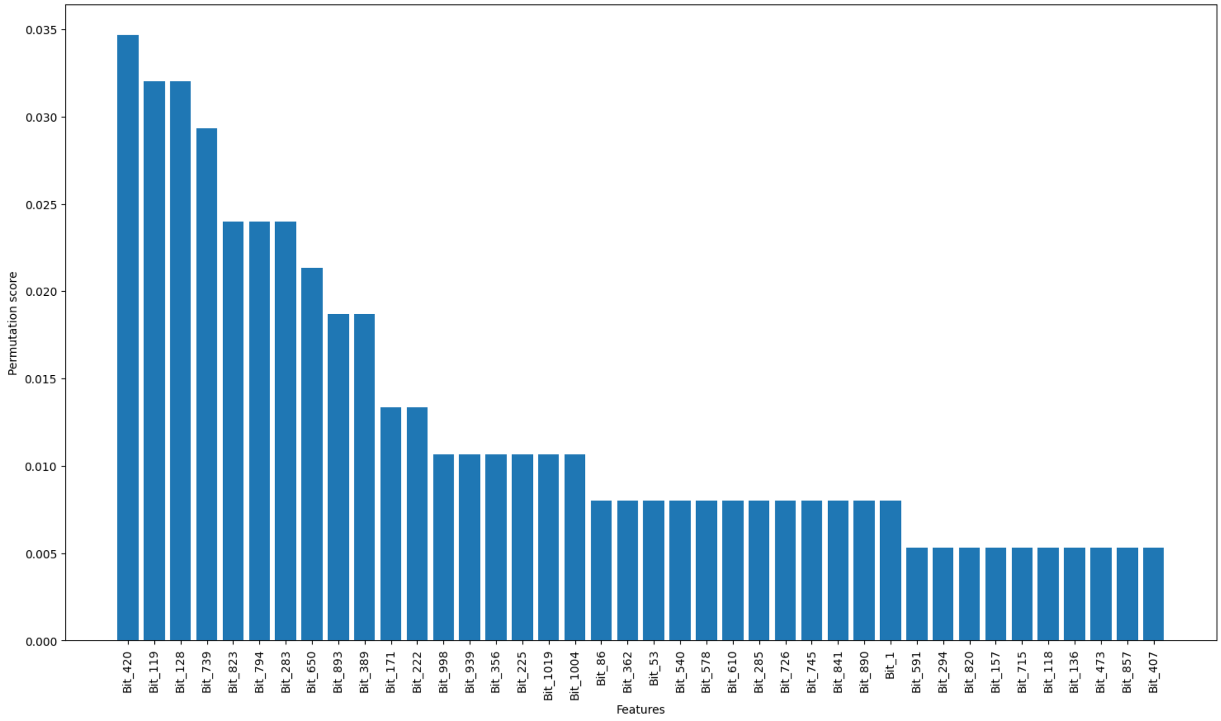

Supplementary Figure 12. Fragments Representing High Permutation Scores in K-Nearest Neighbors Predictor with ECFP. This figure displays fragments corresponding to the 5 bits with the highest Variable Importance (VIP) scores in K-Nearest Neighbors predictors trained with Extended Connectivity Fingerprint (ECFP) for Sour prediction. (Sour prediction has to be taken with care – see discussion)

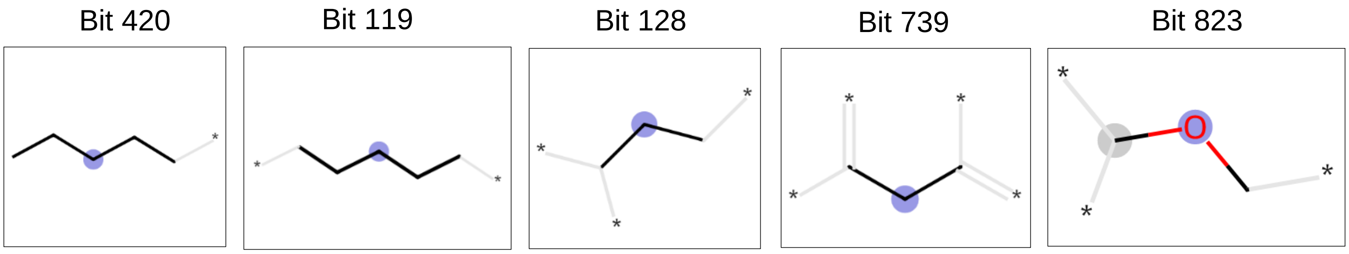

Supplement: Supplementary file 1 [file 13321_2024_935_MOESM1_ESM.pdf]
